# Neglecting the call of the wild: Captive frogs like the sound of their own voice

**DOI:** 10.1371/journal.pone.0181931

**Published:** 2017-07-21

**Authors:** Luiza Figueiredo Passos, Gerardo Garcia, Robert John Young

**Affiliations:** 1 School of Environment and Life Sciences, Peel Building, University of Salford Manchester, Salford, United Kingdom; 2 Chester Zoo, Cedar House, Upton by Chester, Chester, United Kingdom; Universitat Trier, GERMANY

## Abstract

Acoustic communication is highly influential in the expression of social behavior by anuran amphibians, transmitting information about the individual’s physical condition and motivation. We studied the phonotactic (approach movements) responses of wild and captive male golden mantella frogs to conspecific wild and captive playback calls to determine the impact of captivity on social behaviour mediated by vocalisations. Calls were recorded from one wild and two captive populations. Phonotaxis experiments were then conducted by attracting *M*. *aurantiaca* males across a PVC grid on the forest floor or enclosure floor to a speaker. For each playback, the following parameters were recorded to define the accuracy of phonotaxis: (1) number of jumps; (2) jump angles; (3) jump distances; (4) path straightness. During this experiment we observed that wild frogs had a similar behavioural (phonotaxis) response to calls independent of their source while frogs from Chester Zoo had a significantly stronger response to calls of other conspecifics held separately at Chester Zoo. The lack of appropriate phonotaxis response by captive bred frogs to the calls of wild conspecifics could have serious negative conservation implications, if the captive bred individuals were released back to the wild.

## Introduction

Communication is the foundation upon which all social relationships between animals are built [1]. Acoustic communication is probably the most influential trait in the social behavior of anuran amphibians. Although the circumstances in which animals vocalize vary between species, virtually all male frogs incorporate some form of advertisement call into their vocal repertoire that is usually a necessary precursor to successful courtship and mating [2].

In anurans significant information about the individual’s fitness is transmitted by acoustic signals [3,4]. Among male frogs, vocalisations allow the identification of the resource holding potential of an opponent [5,6], facilitate inter-male spacing [7,8] and permit the recognition of territorial neighbours [9]. Field experiments using playback calls have revealed that vocalisations also play an important role in sexual selection during male–male competition and female choice in many species [7,8,10,11].

Phonotaxis is defined as any kind of movement or orientation towards specific acoustic signals [12]. Positive response is taken as evidence of both perception and recognition of the acoustic stimulus by the receiver [9]. It has been widely demonstrated that playback experiments are an adequate methodology to analyse phonotactic responses of frogs [11,12,13].

It is believed that the captive environment can significantly affect the vocalisations of animals to a point where their calls are no longer recognised by wild conspecifics [14]. This would of course have serious implications for reintroduction programmes [14,1]. Therefore, we studied the phonotactic responses of wild and captive male golden mantellas (*Mantella aurantiaca)* to conspecific wild and captive playback calls.

## Methodology

### Study subject

The golden mantella frog (*Mantella aurantiaca)* is a critically endangered species [15], found only in Madagascar with a distribution restricted to a fragment of forest that is under severe threat from mining, agriculture, timber extraction and over-collecting for the pet trade [16]. According to the Amphibian Ark, *ex-situ* assistance is vital for the long-term survival of the golden mantella frog [17].

### Study sites

Golden mantellas calls were recorded from three different populations: wild calls from Mangabe, Madagascar and captive calls from Mitsinjo Captive Breeding Centre (located in Madagascar) and Chester Zoo (UK). The phonotaxis experiments were performed with wild frogs in Madagascar and from captive frogs kept at Chester Zoo.

#### Mangabe area (Madagascar)

Mangabe also known as the “blue forest” is a site of international biodiversity importance, divided into two administrative districts, Moramanga in the north and Anosibe An'ala to the south. Data sampling for this study was done in the Moramanga region. Most breeding ponds for the golden mantellas frogs are found in this area according to recent studies concerning conservation priority sites for mantella frogs.

#### Chester Zoo (UK)

The zoo currently maintains two visually and acoustically isolated *ex situ* groups of *M*. *aurantiaca*, one is on public display at the zoo’s Tropical Realm exhibit from which calls were recorded and a second group is kept off show in a biosecurity container specifically designed for conservation-related research, where the playback experiment was conducted with these frogs. The biosecurity container is kept under temperature and humidity regimes to give the frogs a similar environment as they would experience in the wild. Enclosures are annually modified to keep animals under rainy and dry periods as per their natural environments.

#### Mitsinjo Association Captive Breeding Centre (Madagascar)

This community-run conservation organisation operates around the village of Andasibe in east-central Madagascar and it holds the first Malagasy biosecure facility to protect endangered amphibians. Fifteen local species including a genetically viable population of the golden mantella frog taken from the wild (i.e., genetic founders) collected at the Ambatovy area, and their F1 offspring are currently being kept at Mitsinjo. Only calls from the F1 frogs were recorded and used (no playback experiments were done here).

### Ethical approval

All the research reported in this study was approved by the Chester Zoo’s Ethics Committee, UK and it conforms to all regulations and laws in all relevant countries in relation to care of experimental animal subjects. Furthermore we can confirm, from our post-experimental monitoring, that no animals suffered any injuries, became ill or had their survivorship negatively affected as a result of this study. Furthermore we followed the Association for the Study of Animal Behaviour’s Guidelines for the care of animals [18].

### Recording calls

**Fig 1:**
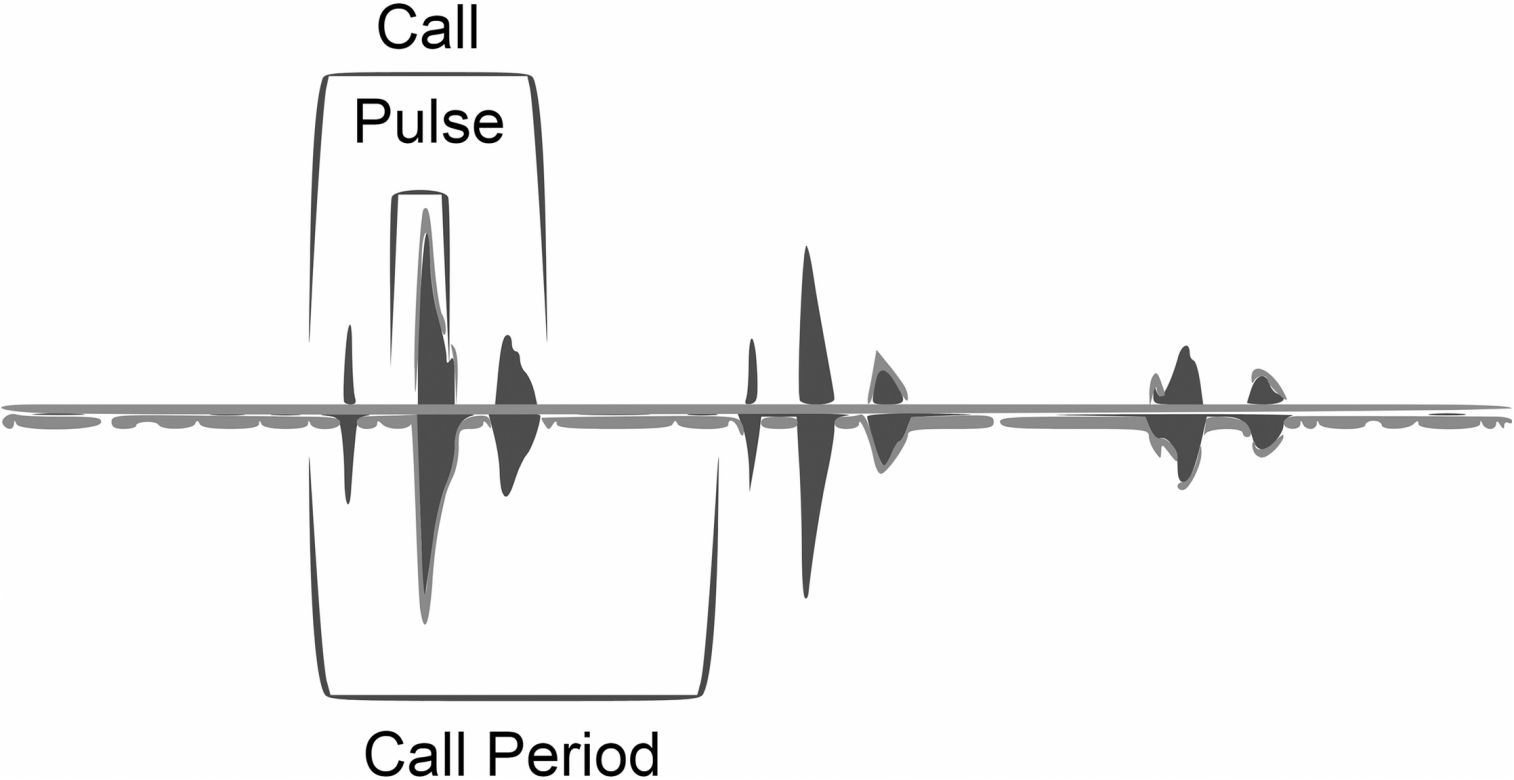
Wild golden mantella frog call waveform showing some measured call characteristics.

Frog calls were recorded using a digital audio recorder (H4n Handheld Digital Recorder, Zoom USA) with an omnidirectional microphone. Before recording calls, a pilot study was undertaken at the University of Manchester with their captive colony of golden mantella frogs to ensure the microphone and recorder had the appropriated sensitivity (i.e. could record all the frequencies emitted by the subjects). Recordings were analysed for call characteristics using Raven software [19]. The characteristics analysed were (Fig 1):

1. Call duration (s): Duration from the beginning of a call to its end.
2. Call period (s): Duration from the beginning of a call to the beginning of the next call.
3. Pulse rate: The number of individual components of each call.
4. Interpulse interval (s): Time between the pulses of a call.
5. Dominant frequency (Hz): The frequency with maximum intensity.

We analysed three call sequences of 20 different males *M*. *aurantiaca* from each population. In addition, to minimize intraspecific variance, we used mean values of the call parameters within and between individuals.

### Phonotaxis experiments

Prior to any experimentation, measurements of sound pressure (noise) levels that animals are already exposed to during routine husbandry at Chester Zoo were taken using a sound pressure meter (SIP95 Sound Level Logging Meter FFT Audio Analyser, Balkon Technology) to avoid exposing animals to any extreme acoustic stimuli. Playback recordings were used with similar amplitude (i.e. volume) to what the animals were already exposed to in captive or natural environments. Calls were previously recorded from the three different populations using a digital audio recorder (H4n Handheld Digital Recorder, Zoom USA) with an omnidirectional microphone. Calls were edited for length and background noise using Audacity® [20] recording and editing software. During the experiment, we recorded the phonotaxis accuracy of a wild (Mangabe) and a captive population (Chester Zoo) of golden mantella frogs to three different recordings (used as treatments): one from a wild population of golden mantellas from Mangabe, and two from captive populations: one from Chester Zoo and one from Mitsinjo. Calls were presented using a randomized block design.

**Fig 2:**
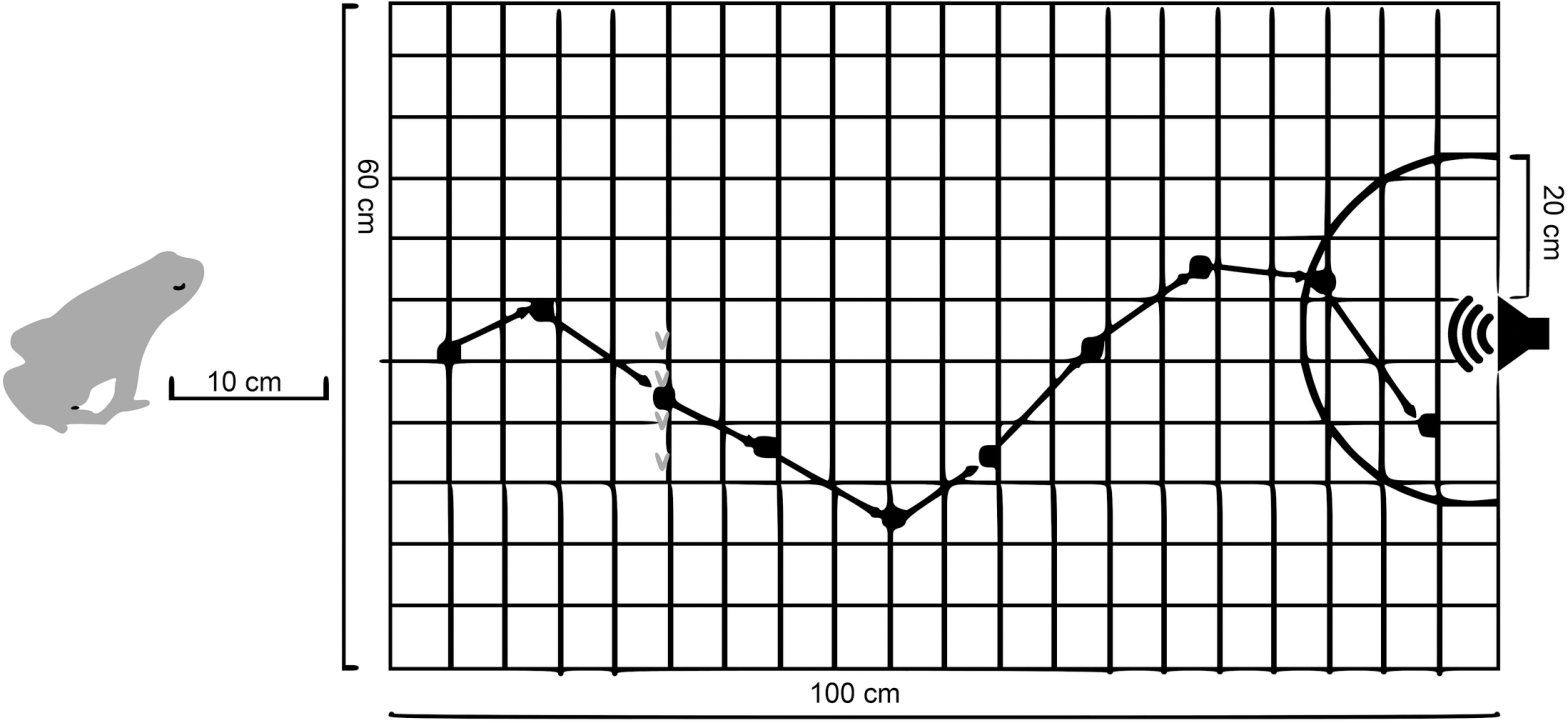
Schematic diagram of a male golden mantella frog when approaching a playback call on a speaker, the grid area is a PVC mat.

Active males were collected by hand from the ponds and put in a plastic box until the experiment. Frogs were kept in the box for nearly one hour, until they had recovered from being hand caught and were behaving normally with no signs of acute stress (i.e. abnormal behaviour, tachycardia). Each animal was tested only once. Phonotaxis playback experiments were than conducted by attracting *M*. *aurantiaca* males across a 100 x 60 cm PVC mat on the forest floor or enclosure floor to a Bluetooth speaker (model HX-P240PK, Jam Plus) broadcasting calls, similar to the method described by Mayer and colleagues (2014). During the experiment, 21 males from Chester zoo and 39 individuals from Mangabe had their phonotaxis response tested. Frogs were placed 10 cm away from the mat (see Fig 2). Trials were not scored if males did not enter the board from the front edge of the board. The experiment was videotaped with a Canon PowerShot SX520 HS digital camera.

Previous playback studies with *Allobates femoralis* [11] and *Ranitomeya imitator* [13] revealed that at distances closer than 30 cm to the sound source the animals searched for a visual signal in addition to the acoustic stimulus; taking this in consideration, playback sessions ended when the frog reached within a perimeter of 20 cm of the speaker (Fig 2).

### Movement analyses

**Fig 3:**
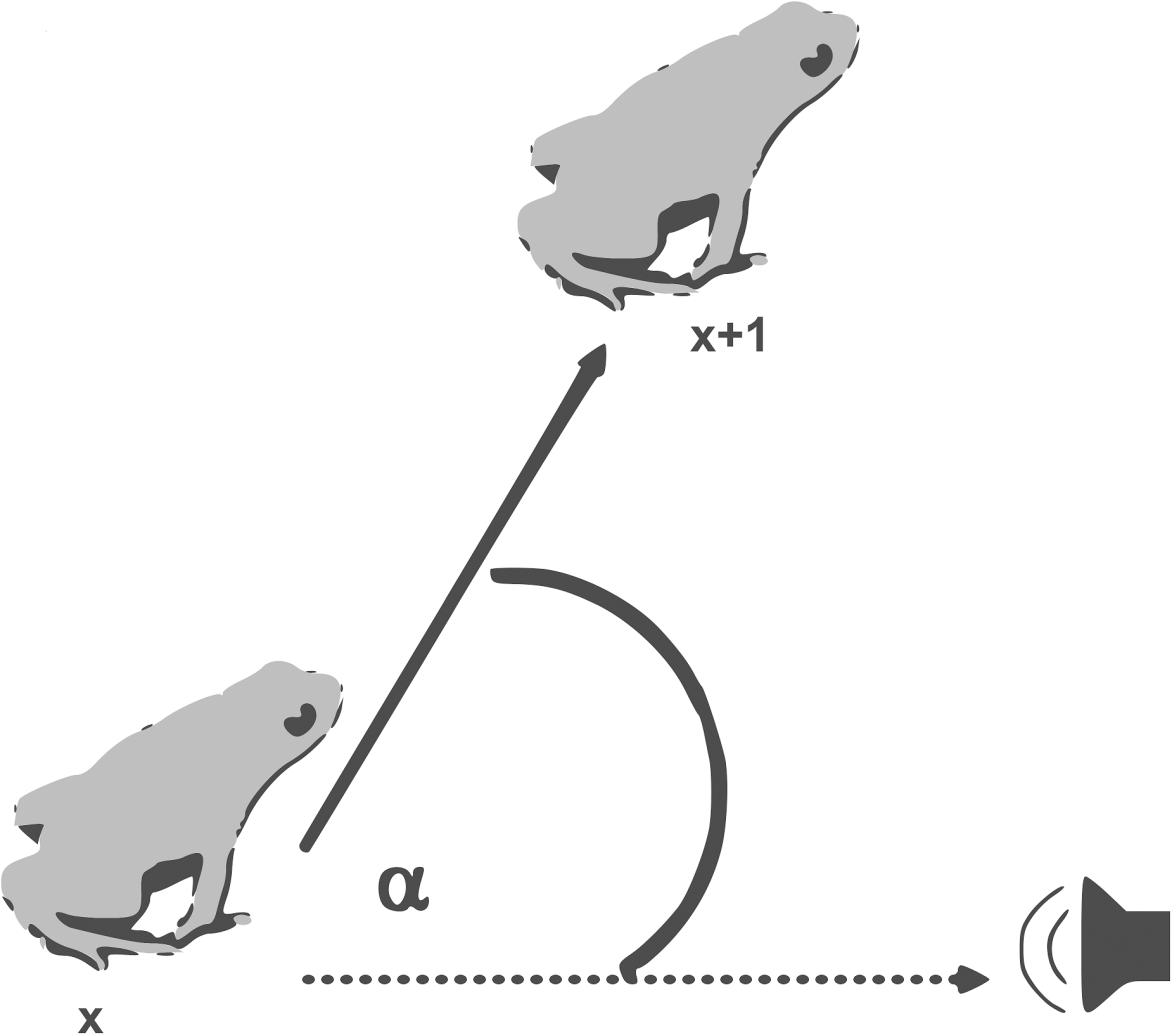
Illustration of how the jump angle α of male golden mantella frogs was calculated in a playback experiment. The dashed line indicates the straight line from the frog to the sound source, X the initial position of the frog and X + 1 the measured jump position.

Each jump of an approaching male was plotted by manually digitizing the recorded videos in a stop-motion view with software called BORIS [21]. The grid on the mat was used to identify frog positions and for calculating distances between positions and jump angles. Jump angles and distances were measured as soon as the animal had entered the board and until it came within 20 cm of the broadcasting speaker (Fig 2). For each playback, the following physical characteristics of frogs were analysed to define the accuracy of phonotaxis: (1) number of jumps; (2) jump angles (jump angle divergence of the new jump position to the target axis; Fig 3); (3) jump distances; (4) path straightness (summing each jump distance for the path taken by the individual in relation to the straight line from the first entered position to the target); (5) duration (how long, in seconds, the frogs took to reach the speaker). The accuracy of the phonotactic approach was quantified using jump angles and the straightness of the path; values are given as percentage of path length in relation to the straight-line distance. All statistical analyses were done using R Studio [22].

## Results

Call characteristics (Table 1) were compared between the three different populations using one-way ANOVA tests. Tests found significant differences between the populations on all the parameters analysed (p<0.05). The Tukey *posthoc* test (Table 2) confirmed that calls from Chester Zoo animals were significantly different (p<0.05 in all cases) from calls obtained from the wild population on all the analysed characteristics. Vocalisations from Mitsinjo breeding centre were significantly different from Mangabe calls in duration and period (p<0.05). Chester Zoo and Mitsinjo recording were statistically different in all parameters except for pulse numbers (p<0.05).

**Table 1:** Call characteristics results for different wild and captive populations of golden mantella frogs.

Population	Origen	Duration (s)	Period (s)	Pulse rate	Interpulse (s)	Dominant frequency(Hz)
		± sd	± sd	± sd	± sd	± sd
Mangabe	Wild	0.043±0.004	0.09±0.05	2.92±0.27	0.008±0.002	4875±0.00
Chester Zoo	Captive	0.033 ±0.011	0.75±0.620	3.9±0.72	0.01±0.006	5198.01±172.84
Mitsinjo	Captive	0.062±0.008	0.12±0.063	4.04±0.19	0.005±0.001	4941.96±146.25

sd = standard deviation

**Table 2:** *Posthoc* Tukey test results for golden mantella frogs’ call characteristics from different wild and captive populations.

Populations	Duration	Period	Pulse rate	Interpulse	Dominant Frequency
Mangabe x Mitisnjo	p< 0.01	ns	p< 0.01	ns	ns
Mangabe x Chester	p< 0.01	p< 0.01	p< 0.01	p<0.05	p< 0.01
Mitisnjo x Chester	p< 0.01	p< 0.01	ns	p< 0.01	p< 0.01

**Fig 4:**
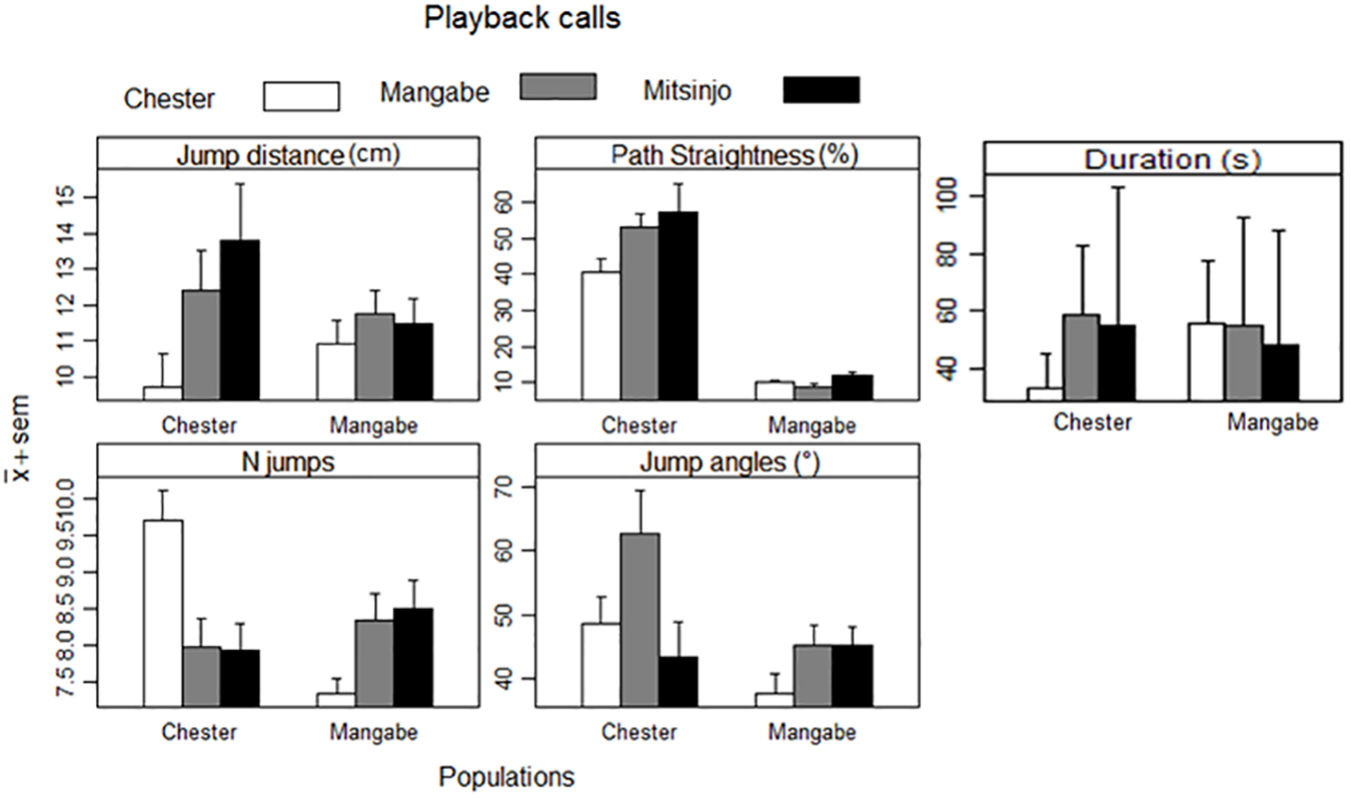
Summary of phonotactic movement results (mean +Standard Error of the Mean) of golden mantilla frogs towards playback calls.

Phonotactic experiments resulted in 34 approaches of wild golden mantellas and 21 for the Chester Zoo’s frogs (i.e. a total of 55 different individuals). In general, captive frogs took longer and used a lengthier and less accurate path to reach the speaker than wild frogs. All trials with Chester Zoo’s frogs resulted in a phonotaxis response, however, five trials (two with Mitsinjo’s calls, two with Chester’s calls and one for Mangabe’s calls) from Mangabe’s animals, had no phonotaxis response (i.e. no movement) and were, therefore, not analysed. All successful trials were scored for number of jumps, jump distances, jump angles, path straightness and duration (Fig 4).

Generalised linear mixed models (GLMM) were used to compare the golden mantella frogs’ phonotactic movement in response to different playback treatments (see Table 3). Calls were used as fixed factors and location as random factors. Wild individuals’ responses to wild calls were used as the species’ natural response and, this was considered as a reference for an expected reaction towards conspecifics. The wild frogs from Mangabe showed no difference (p>0.05) in any of the variables measured for all of the three calls (i.e., wild, or captive) used during the experiment.

**Table 3:** Parameter estimates for the Generalized Linear Mixed Models describing the relationship between playback treatment (call sources) and analysis of phonotaxis response of male golden mantella frogs.

Population	Call	N	Parameter	Coefficient	p-value
Chester	Mangabe	7	N jumps	-0.04	ns
Chester	Mangabe		Jump angles	17.3	0.004
Chester	Mangabe		Jump distance	0.79	ns
Chester	Mangabe		Path straightness	39.9	0.006
Chester	Mangabe		Duration	10.59	ns
Chester	Mitsinjo	7	N jumps	-0.04	ns
Chester	Mitsinjo		Jump angles	-2.78	ns
Chester	Mitsinjo		Jump distance	2.29	ns
Chester	Mitsinjo		Path straightness	47.1	<0.001
Chester	Mitsinjo		Duration	6.39	ns
Chester	Chester	7	N jumps	0.09	<0.001
Chester	Chester		Jump angles	3.49	<0.001
Chester	Chester		Jump distance	-1.8	ns
Chester	Chester		Path straightness	32.2	0.024
Chester	Chester		Duration	7.08	<0.001
Mangabe	Mangabe	13	N jumps	-0.02	ns
Mangabe	Mangabe		Jump angles	1.27	ns
Mangabe	Mangabe		Jump distance	0.18	ns
Mangabe	Mangabe		Path straightness	-2.43	ns
Mangabe	Mangabe		Duration	6.43	ns
Mangabe	Mitsinjo	13	N jumps	2.15	ns
Mangabe	Mitsinjo		Jump angles	4.98	ns
Mangabe	Mitsinjo		Jump distance	1.53	ns
Mangabe	Mitsinjo		Path straightness	2.47	ns
Mangabe	Mitsinjo		Duration	7.8	ns
Mangabe	Chester	13	N jumps	-0.13	ns
Mangabe	Chester		Jump angles	1.27	ns
Mangabe	Chester		Jump distance	-0.58	ns
Mangabe	Chester		Path straightness	-2.73	ns
Mangabe	Chester		Duration	9.19	ns

Chester Zoo’s frogs had significant differences (p<0.05) in the number of jumps and duration to the speaker when their own call was presented, jump angles for Mangabe and zoo calls, and path straightness between all calls (Table 4); however, different calls had no impact on jump distance (p>0.05). Despite frogs making a significantly higher number of jumps to reach the target, phonotaxis accuracy was higher for calls recorded at Chester Zoo with a straighter, shorter and faster path to the speaker (Fig 4). Path straightness when Mangabe’s calls were played, resulted in a longer path in relationship to the path used during Chester Zoo calls, and an even longer path was used for Mitisinjo’s playback calls.

**Table 4:** T-test results of the movement analysis of phonotaxis response between wild and captive golden mantella frogs.

Location	Parameter	Mean	SEM	t	N	p-value
Wild	N jumps	8.04	0.18	1.97	55	0.02
Captive	N jumps	8.64	0.23			
Wild	Jump angles (°)	51.79	3.17	2.54	55	0.04
Captive	Jump angles (°)	42.62	1.72			
Wild	Jump distance (cm)	11.74	0.68	0.47	55	0.55
Captive	Jump distance (cm)	11.37	0.38			
Wild	Path straightness (%)	49.44	0.45	12.09	55	0.001
Captive	Path straightness (%)	10.33	2.99			
Wild	Duration (s)	49.18	2.00	3.15	55	0.001
Captive	Duration (s)	60.11	2.83			

When the responses of both populations were compared using a t-test (Table 4) all the parameters were statistically different (p<0.05), except for jump distance. Wild frogs had a straighter, shorter and faster route even though they made shorter jumps (Fig 4).

## Discussion

The analysis of different call parameters showed that calls from Chester Zoo’s frogs were statistically different from wild frogs’ vocalisations in all analysed characteristics. Whereas the call analyses from the colony held at Mitsinjo breeding centre showed greater similarities with the wild conspecifics. The implication of the observed differences could be negative in terms of reproduction if captive frogs were to be released to the wild. The breeding behaviour of golden mantella frogs involves males calling to court the females with multiple males vocalising simultaneously [23]. Males with calls modified by captivity, if reintroduced could have their ability to attract females compromised.

Vocalisations are moulded by the acoustic environment in which the species is found [24,25]. A zoo’s environment has different background noises from sources such as heaters, air filters and visitors, which will lead to a different acoustic complexity (soundscape) than wild habitats. It has already been proved that anthropogenic sounds can alter the calling behaviour of anurans by causing males to modulate their call rate or call frequency [1,25]. Animals being kept in captivity for many generations could have their calls significantly affected by their environment, while frogs that have been in captivity for only one generation, would not be so affected. This would explain the results found on the call parameters of the Mitsinjo frogs, which had greater similarities with wild calls, while Chester Zoo animals had calls that were significantly different.

During the phonotaxis experiment we observed that wild frogs had a similar behavioural (phonotaxis) response to calls of conspecifics independent of their source (i.e. wild versus captive) while frogs from Chester Zoo had a significantly stronger response to their own calls. Wild frogs had more accurate response, reaching the speaker using a shorter path and in less time while captive frogs were using a longer path and more time, even though they had longer jumps. It is important to notice that wild frogs would recognize and react in a similar way to captive frogs despite the changes found in their calls. Captive frogs had a weak response to wild calls and, if captive frogs are not able to recognize wild calls or respond appropriately, this could, potentially have negative consequences[26, 27], such as assortative mating among released individuals, with females only being attracted to captive males, leading to two genetically disconnected populations [28]. This could, potentially, decrease the conservation value of the reintroduction programme.

The golden mantella frogs breeding behaviour is characterized by groups of males competitively calling to attract females; in this scenario it is usual to observe males showing aggressive behaviour toward other males as a sign of competition for females. This aggressive behaviour have been describe in the wild and observed in captive populations [28]. The phonotactic response observed in wild frogs corroborate with this premise, while captive frogs only showed this response to their own calls.

Species recognition is a fundamental problem for animals in social contexts [26] for a reintroduction to be successful, released individuals must survive and breed [27, 28].Although the accuracy of phonotaxis does not necessarily reflect the accuracy of perception, movement analysis is a powerful approach to examine the auditory abilities of animals [29]. When the responses of the two populations were compared, it was possible to observe that frogs from Mangabe (wild) showed a more precise phonotaxis response to calls than golden mantella frogs kept in captivity. Wild male golden mantella frogs would react to defend their territory against all possible opponents presented during the playback experiment, implying that they would recognize conspecific calls even from captive populations.

Animals in captivity are in a confined space in close proximity to other males [30], which could lead to overlapping territories and to recognition of individuals as neighbors and not as threats (i.e. “dear enemies”; [31]). This would explain the differences observed during the phonotaxis experiment, with captive animals using a longer and less accurate path and, taking longer to reach the speaker. Social recognition is thought to enhance fitness by providing a mechanism that allows animals to direct appropriate behaviours toward specific individuals during repeated social interactions, “the dear enemy effect” [32]. Evidence for the dear enemy effect typically consists of a relatively lower level of aggression exhibited by territory holders toward neighbours [32]. Dear enemy relationships, however, are not common among territorial species, and several studies have reported that territory residents respond similarly to neighbours and strangers under some conditions [31].

Frogs characteristically avoid moving unless totally necessary, since it is both energetically costly and increases predation risk [4]. The receiver of an acoustic signal has to judge the sender’s motivational state and adjust his own reaction according to the costs [29]. If calls are not perceived as intruders, but as neighbours, it would not trigger such a phonotaxis response. The decision to approach and chase an intruder is, therefore, influenced by the trade-off between fitness costs and benefits [29].

Animals may adjust the characteristics of their vocalizations in response to temporary changes in the background noise [23,1]. Such short‐term vocal adaptations have been examined in insects, anurans, birds, and mammals [1]. Pre-release training associated with a soft release programme, could help re-shape calls from captive animals to increase their chances of breeding in the wild. Similar approaches have been used successfully in golden lion tamarins (*Leontopithecus rosalia*) [31].

Communication can be crucial for breeding success in golden mantella frogs if individuals are being bred for conservation; it is of critical importance to make sure that captive animals, if released, will have the same chances of breeding as their wild counterparts. Captive breeding is growing as an indispensable tool in conservation tool for many species [33], especially amphibians. However, it is important to fully understand the impact of captivity on a species’ behaviour before releasing individuals back into the wild.
